# Dual-Functional Antioxidant and Antiamyloid Cerium Oxide Nanoparticles Fabricated by Controlled Synthesis in Water-Alcohol Solutions

**DOI:** 10.3390/biomedicines10050942

**Published:** 2022-04-19

**Authors:** Katarina Siposova, Veronika Huntosova, Ivana Garcarova, Yuliia Shlapa, Illia Timashkov, Anatolii Belous, Andrey Musatov

**Affiliations:** 1 Department of Biophysics, Institute of Experimental Physics, Slovak Academy of Sciences, Watsonova 47, 04001 Kosice, Slovakia; garcarova@saske.sk (I.G.); musatov@saske.sk (A.M.); 2 Center for Interdisciplinary Biosciences, Technology and Innovation Park, P.J. Safarik University in Kosice, Jesenna 5, 04154 Kosice, Slovakia; 3 Vernadskii Institute of General and Inorganic Chemistry of NAS of Ukraine, Palladina Ave., 32/3403142, 03142 Kyiv, Ukraine; yuliashlapa@ukr.net (Y.S.); timashkov@ukr.net (I.T.); agrbilous@ukr.net (A.B.)

**Keywords:** cerium oxide nanoparticles, antiamyloid activity, oxidative stress, nanozymes, bioactivity

## Abstract

Oxidative stress is known to be associated with a number of degenerative diseases. A better knowledge of the interplay between oxidative stress and amyloidogenesis is crucial for the understanding of both, aging and age-related neurodegenerative diseases. Cerium dioxide nanoparticles (CeO_2_ NPs, nanoceria) due to their remarkable properties are perspective nanomaterials in the study of the processes accompanying oxidative-stress-related diseases, including amyloid-related pathologies. In the present work, we analyze the effects of CeO_2_ NPs of different sizes and Ce^4+^/Ce^3+^ ratios on the fibrillogenesis of insulin, SOD-like enzymatic activity, oxidative stress, biocompatibility, and cell metabolic activity. CeO_2_ NPs (marked as Ce1–Ce5) with controlled physical–chemical parameters, such as different sizes and various Ce^4+^/Ce^3+^ ratios, are synthesized by precipitation in water–alcohol solutions. All synthesized NPs are monodispersed and exhibit good stability in aqueous suspensions. ThT and ANS fluorescence assays and AFM are applied to monitor the insulin amyloid aggregation and antiamyloid aggregation activity of CeO_2_ NPs. The analyzed Ce1–Ce5 nanoparticles strongly inhibit the formation of insulin amyloid aggregates in vitro. The bioactivity is analyzed using SOD and MTT assays, Western blot, fluorescence microscopy, and flow cytometry. The antioxidative effects and bioactivity of nanoparticles are size- or valence-dependent. CeO_2_ NPs show great potential benefits for studying the interplay between oxidative stress and amyloid-related diseases, and can be used for verification of the role of oxidative stress in amyloid-related diseases.

## 1. Introduction

The deposition of protein amyloid aggregates is associated with several degenerative diseases, including Alzheimer’s disease, Parkinson’s disease, and diabetes mellitus. There are numerous hypotheses on how the accumulation of amyloid aggregates is involved in cell and tissue degradation [1,2,3,4]. For example, detailed studies have described a functional link between protein amyloid formation and oxidative stress, and have revealed that oxidative stress can be either causative or consecutive to protein aggregation [5,6,7]. In fact, reactive oxygen species (ROS) can damage the key cellular components, such as the DNA, phospholipids, and proteins, which ultimately can lead to cell death; therefore, they play an important role in the pathogenesis of many diseases [6,8,9]. Some of the reports suggest that oxidative stress can be responsible for the formation of protein amyloid aggregates. It was demonstrated that oxidative stress-induced oxidation or modification of amino acids can lead to protein aggregation [5,10,11,12]. On the contrary, several studies showed that aggregation can generate oxidative stress or protect proteins against oxidative damage [12,13,14,15,16]. Therefore, a better knowledge of the interplay between oxidative stress and amyloidogenesis is essential for the understanding of both aging and age-related neurodegenerative diseases.

Many proteins can form intracellular or extracellular aggregates. It was suggested that the ability to form cross-β-sheet-rich amyloid fibrils may be a common property of the polypeptides [1,2,4]. Insulin is a disulfide-linked small globular protein consisting of two polypeptides, the A chain and the B chain. Insulin is one of the proteins able to form amyloid fibrils, especially under denaturing conditions, such as elevated temperatures, low pH, and agitation [17,18,19,20]. Moreover, it is documented that insulin amyloid aggregation is linked to many pathological processes [20], since insulin aggregates were identified in vivo near the sites of insulin injection [21,22,23]. The physiological and therapeutic importance of insulin, and the fact that insulin easily self-associates in vitro, have made this globular protein an outstanding model for protein amyloid aggregation studies.

Nanosized materials have been and are currently being extensively investigated for their potential medical uses, including drug delivery, medical imaging, antibacterial, and antiviral applications. Among the various nanosized substances, cerium dioxide nanoparticles (CeO_2_ NPs), due to their remarkable properties, attract much interest in the study of the processes accompanying oxidative stress-related diseases. Attractive physical–chemical properties of cerium are derived from shielded 4f-electrons in the electronic configuration of [Xe]4f26s2. In addition, the low redox potential of the Ce^4+^/Ce^3+^ redox couple (~1.52 V) allows the coexistence of both oxidation states III and IV [24,25,26]. Furthermore, the size of the synthesized nanoparticles can be controlled during synthesis [27,28,29]. Importantly, the Ce^4+^/Ce^3+^ ratio on the NPs’ surfaces affects their catalytic activity. The low toxicity of CeO_2_ NPs ensures the relative safety of its use in vivo, which makes it possible to consider this material as a potential drug for the treatment of some diseases primarily associated with oxidation stress, including age-related neurodegenerative diseases [30,31]. It should also be mentioned that because of the unique ability of CeO_2_ NPs to switch oxidation states between III and IV, CeO_2_ NPs were successfully used as biosensors for the detection of hydrogen peroxide, glucose, and other bioactive compounds and biomolecules [32,33,34]. Previously, we have demonstrated the antiamyloidogenic effect of uncoated, non-agglomerated crystallites of CeO_2_ NPs with average sizes of 5–6 nm synthesized by precipitation from reversal microemulsions based on the non-ionic detergent Triton-X-100 [35]. However, the synthesis of CeO_2_ NPs by precipitation in reversal microemulsions is rather complicated in terms of implementation and also expensive, since the yield of synthesized NPs is quite low. Recently, we successfully prepared and characterized a series of CeO_2_ NPs with controllable sizes and Ce^4+^/Ce^3+^ ratios using a method of precipitation in water–alcohol solutions [36].

In the present work, we analyzed the effects of CeO_2_ NPs of different sizes and Ce^4+^/Ce^3+^ ratios on the fibrillogenesis of insulin and the pseudo-enzymatic activity by monitoring the SOD activity, as well as the impacts on oxidative stress, biocompatibility, and cell metabolic activity. The results revealed the antiamyloid effects of all tested CeO_2_ NPs on insulin amyloid fibril formation. Moreover, the NPs exhibited significant antioxidant activity through regulating the oxidant and antioxidant cell balance. Therefore, the results suggest that CeO_2_ NPs can be used in future experiments to prove or disprove the hypothesis that oxidative stress may play a role in the modulation of protein amyloid formation.

## 2. Materials and Methods

### 2.1. Synthesis and Characterization of CeO_2_ NPs

A set of 5 CeO_2_ NPs (marked as Ce1, Ce2, Ce3, Ce4, and Ce5) was synthesized by precipitation in the water–alcohol (bi-distilled water and isopropanol (IPA)) solutions, where the concentrations of alcohol varied from 0 to 90%. Briefly, a water–alcohol solution of ammonium was added to the solution of cerium (III) nitrate with constant stirring. The obtained NPs were centrifugated and washed with ethanol and bi-distilled water. CeO_2_ NPs were characterized using physical methods of analysis: crystallographic properties such as the degree of crystallinity were studied using the X-ray diffraction method (XRD), the chemical states of the surface of CeO_2_ NPs (namely the percentage of Ce^3+^) were investigated via X-ray photoelectron spectroscopy (XPS), and the stability of the synthesized NPs in the aqueous suspensions was studied via dynamic light scattering (DLS). A detailed description of CeO_2_ NP synthesis and characterization via the above-mentioned techniques was published recently [36].

To study the morphology of CeO_2_ NPs, a drop of diluted CeO_2_ suspension was deposited onto the copper grid with the formvar film and air-dried. Micrographs of CeO_2_ NPs were recorded using a TEM JEOL JEM 1230 transition electron microscope. The particles sizes and their distributions were calculated using Image Tools and Origin 9.0 software packages (OriginLab Corp., Northampton, MA, USA).

### 2.2. Antiamyloidogenic Effects of CeO_2_ NPs on Insulin Amyloid Fibrillization

Insulin (human recombinant, expressed in yeast, 12643; Sigma-Aldrich, Inc., St. Louis, MO, USA) was dissolved in 100 mM NaCl solution, pH 1.6 (hereinafter referred to as NaCl solution), to a final concentration of 25 μM. The solution was incubated in an Eppendorf comfort thermomixer at 65 °C for 2 h under constant agitation (500 or 1200 rpm). The formation of insulin amyloid fibrils was monitored by fluorescence (Thioflavin T (ThT, T3516; Sigma-Aldrich, Inc., St. Louis, MO, USA)) and 1-anilinonaphyhalene-8-sulfonic acid (ANS, A1028; Sigma-Aldrich) assays and confirmed by atomic force microscopy (AFM).

To evaluate the antiamyloid activity of CeO_2_ NPs, two different strategies were used: (I) investigation of the concentration-dependent effects of CeO_2_ NPs; (II) monitoring of the time-dependent effects of CeO_2_ NPs on insulin self-association. Within dose-dependent experiments, we tested the ability of nanoparticles: (i) to affect insulin amyloid formation (inhibition of amyloid formation); (ii) to disassemble preformed amyloid fibrils, following recently described protocols [35,37,38]. For the dose-dependent analysis, the aliquots of CeO_2_ NPs samples (from freshly prepared stock solutions in ultrapure H_2_O) were added: (a) to 25 μM of insulin in NaCl solution at protein-to-CeO_2_ NPs ratios ranging from 1:0.01 to 1:10 (weight to weight ratio), with samples incubated at 65 °C for up to 2 h under constant agitation (500 or 1200 rpm) (inhibition measurements); (b) to 25 μM of preformed insulin fibrils in NaCl solution followed by incubation for 24 h at 37 °C (disassembling activity). ThT or ANS was added to the protein samples (5 μM of insulin) at a final concentration of 25 μM followed by incubation for 1 h at 37 °C. The fluorescence intensity measurements were performed in triplicate using a Synergy Mx (BioTek Inc., Crawfordsville, IN, USA) spectrofluorometer in a 96-well plate with excitation wavelengths set at 440 nm and 380 nm for ThT and ANS, respectively. The emission was recorded at 485 nm. The emission and excitation slits were set at 9.0/9.0 nm and the top probe vertical offset was 6 nm. The fluorescence intensities of samples were normalized to the fluorescence intensities of amyloid insulin aggregates prepared in the absence of NPs (taken as 100%). The error bars represent the average deviation for repeated measurements of three separate samples. The experimental data were fitted by varying four parameters in the sigmoidal logistic equation using SigmaPlot version 14.0 (Systat Software Inc.; Erkrath, Germany) as described previously [38]. The IC_50_ (the CeO_2_ NP concentration leading to 50% inhibition of amyloid fibrillization) and DC_50_ (the CeO_2_ NP concentration leading to 50% disassembly of pre-formed fibrils) values were calculated from the dose–response inhibition and disassembly curves, respectively.

In the next set of experiments, the effects of CeO_2_ NPs on the kinetics of insulin amyloid formation were assessed. For kinetic measurements, the aliquots of insulin solution incubated at 65 °C in the absence and presence of the desired concentration of CeO_2_ NPs were withdrawn at varying times, mixed with ThT, incubated at 37 °C for 1 h, and analyzed as described above. The experimental data were fitted using a 4-parameter sigmoidal logistic equation using SigmaPlot version 14.0 (Systat Software Inc.).

### 2.3. Atomic Force Microscopy

AFM is used for analyzing the morphology of formed amyloid aggregates alone and in the presence of CeO_2_ NPs, as well as for examination of the disassembly potential of CeO_2_ NPs. Samples analyzed using AFM were prepared by casting 10 μL aliquots on a freshly cleaved mica surface (the highest grade V1 mica discs, Ted Pella, Inc., Redding, CA, USA). After adsorption to the surface (5–10 min at 25 °C), the mica surface was washed with ultrapure water (18.2 MΩ cm) and the samples were dried under a stream of nitrogen. The AFM images were obtained using a scanning probe microscope (Veeco di Innova, Bruker AXS Inc., Madison, WI, USA) working in tapping mode. The scan rate range was 0.5–0.75 kHz. The resolution of the images was 1024 × 1024 pixels/image. All images are presented without smoothing or noise reduction. The AFM images were analyzed using NanoScope Analysis1.20 (Veeco di Innova, Bruker AXS Inc., Madison, WI, USA).

### 2.4. Pseudo-Enzymatic, Superoxide Dismutase Activity

The superoxide dismutase (SOD) activity was assessed via a colorimetric assay using an SOD determination kit (SIGMA-Aldrich, 19160) that utilized WST-1 (2-(4-iodophenyl)-3-(4-nitrophenyl)-5-(2,4-disulfophenyl)-2H-tetrazolium, a monosodium salt, which produces the water-soluble formazan dye upon reduction by superoxide anion. Briefly, 20 µL of CeO_2_ NPs dispersed in Mili-Q water was added to a well of a 96-well plate and mixed with 200 µL of WST 1. The reaction was started by the addition of 20 µL xanthine oxidase solution to the reaction mixture. After 20 min of incubation at 37 °C, the absorbance at 450 nm was measured using a microplate reader (Synergy BioTek). The SOD-like activity, A_SOD_ (inhibition rate, %), was calculated using the following Equation (1):

(1) $$\mathrm{SOD}\;\mathrm{activity}\;(\mathrm{inhibition}\;\mathrm{rate}\;\%)=\left\{\frac{\left[\left(A_{\mathrm{blank}1}-{\;A}_{\mathrm{blank}3}\right)-\left(A_{\mathrm{sample}}-{\;A}_{\mathrm{blank}2}\right)\right]}{\left({A\;}_{\mathrm{blank}1}–{\;A}_{\mathrm{blank}3}\right)}\right\}*100$$

### 2.5. Cell Culture

U87 MG human glioma cells (Cells Lines Services, Eppelheim, Germany) were grown in a complete cell culture medium. Dulbecco’s modified Eagle medium (D-MEM, Gibco Invitrogen, Life Technologies Ltd., Waltham, MA USA) was supplemented with 10% fetal bovine serum (FBS, Gibco Invitrogen, Life Technologies Ltd.). The medium contained L-glutamine (862 mg/L), sodium pyruvate (110 mg/L), glucose (4500 mg/L), and penicillin/streptomycin (1% *w*/*w*). Cells were dark-cultivated at 5% CO_2_ in a humified atmosphere.

### 2.6. MTT Assay

A metabolic assay on cell mitochondria was based on the transformation of 3-(4,5-dimethylthiazol-2-yl)-2,5-diphenyltetrazolium bromide (MTT, Sigma-Aldrich, Taufkirchen, Germany) to purple formazan. The formazan (Sigma-Aldrich, Germany) production was measured in a 96-well plate reader (GloMax^®^-Multi + Detection System with Instinct Software, Promega Corporation, Madison, WI, USA). Cells were incubated for 24 h with Ce1 and Ce5 at concentrations of 50–400 µg/mL. The level of significance was evaluated with a one-way ANOVA test.

### 2.7. Microscopic Visualization of Cells

Cells were visualized in bright field and fluorescence modes using an inverted LSM700 confocal microscope (Zeiss, Jena, Germany) equipped with a 40× water immersion objective and a CCD camera (AxioCam HRm, Zeiss, Germany). The fluorescence of the samples was collected after excitation with an Hg lamp light filtered with appropriated filters (classical fluorescence images) or solid-state lasers at 405, 488, and 555 nm (confocal fluorescence images). Nuclei were stained by 10 µg/mL Hoechst 33,258 (ThermoFisher Scientific, Waltham, MA, USA), with excitation at 405 nm and emissions at 450 ± 40 nm in confocal fluorescence microscopy mode using an FS02 filter cube (ZEISS, excitation G 365, beam splitter FT 395, emission LP 420) for classical fluorescence microscopy. Lysosomes were stained with 200 nM LysoTracker Green (ThermoFisher Scientific, USA), with excitation at 488 nm and emissions at 500–540 nm using an FS10 filter cube (ZEISS, excitation BP = 450–490, emission BP = 515–565). The size of the lysosomes was measured using ImageJ software (National Institutes of Health; Bethesda, MD, USA). Cellular reactive oxygen species (ROS) were detected with the DCFDA/H2DCFDA cellular ROS assay kit (ab113851, Abcam, UK) via confocal fluorescence microscopy using 20 µM DCFDA (excitation at 488 nm and emission 500–540 nm). The mitochondrial membrane potential was visualized with 10 nM tetramethylrhodamine methyl ester (TMTM, Sigma-Aldrich, Darmstadt, Germany) with 555 nm excitation and emissions > 560 nm. Reduced glutathione (GSH) was detected by 20 µM ThiolTracker^TM^ Violet (T10095, ThermoFisher Scientific, USA) staining using fluorescence confocal microscopy (excitation at 405 nm and emissions at 500–540 nm).

### 2.8. Flow Cytometry

U87 MG cells were incubated for 24 h with insulin fibrils, Ce1, and Ce5 at concentrations of 45 and 375 µg/mL. Cells were detached before measurement and labeled for 30 min using Thiol Tracker Violet to detect GSH levels in cells. Cell populations were detected via flow cytometry (MACSQuant^®^Analyzer, Miltenyi, Germany) in a V1 (excitation 405 nm, emission 450/50 nm) channel and divided into four quadrants according to Thiol Tracker Violet fluorescence intensities.

## 3. Results and Discussion

### 3.1. Synthesis of Nanoparticles and Their Characterization

**Figure 1:**
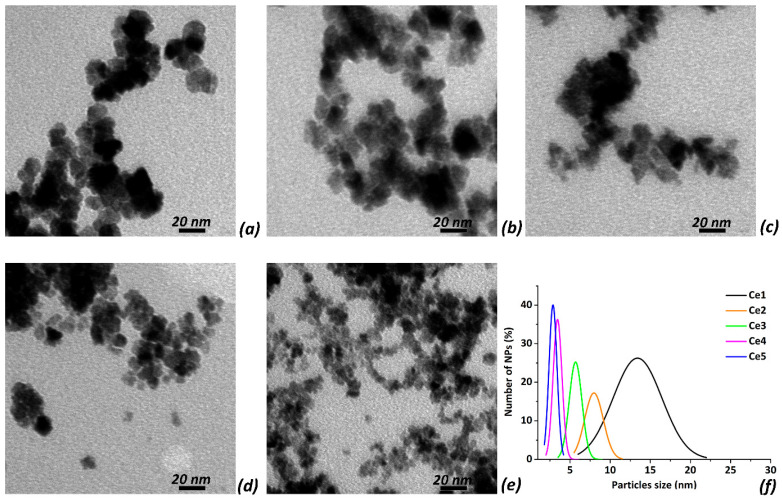
Representative TEM images of CeO_2_ NPs Ce1 (**a**), Ce2 (**b**), Ce3 (**c**), Ce4 (**d**), and Ce5 (**e**), and normalized curves of the particle size distributions (**f**).

A set of five CeO_2_ NPs (marked as Ce1, Ce2, Ce3, Ce4, and Ce5) was synthesized via precipitation in water–alcohol solutions, as we reported recently [36]. According to published XRD data, synthesized CeO_2_ NPs were single-phase, had a crystalline structure with the space group of *Fm3m*, with particle sizes ranging from ~15 nm (Ce1 NPs) to ~6 nm (Ce5 NPs) and the degrees of crystallinity ranging from 80% to 65%, respectively. The obtained data demonstrated that the crystalline size of CeO_2_ NPs decreased with the increase in alcohol percentage in the reaction mixture used for the precipitation [36]. In the present work, detailed investigations of the morphology of the synthesized CeO_2_ NPs were performed using transmission electron microscopy (TEM), and representative TEM images are shown in Figure 1a–e. The calculated average particles sizes are summarized in Table 1. NPs were non-agglomerated and their sizes varied in the range of ~3 to ~14 nm. Figure 1f represents the normalized curves of size distributions for Ce1–Ce5 NPs. According to these curves, increasing the alcohol concentration in the reaction mixture resulted in the synthesis of CeO_2_ NPs with smaller sizes and narrower size distributions. The NP sizes observed via TEM for the smallest CeO_2_ NPs were quite different from the XRD published data. We believe that this difference was because the XRD method calculates the averaged crystalline diameter of NPs while the TEM also obtains the size distributions. Therefore, the insignificant number of the large-sized particles may have increased the XRD diameter compared to the TEM data. The obtained data also showed that the percentage of Ce^3+^ ions on the surface of CeO_2_ NPs increased with decreasing NP size. The behavior of the obtained CeO_2_ NPs in the aqueous suspensions, namely their stability, was studied via DLS analysis (detailed experimental data were reported recently [36]). The measured zeta potential values for a set of Ce1–Ce5 NPs were higher than ζ = +40 mV, suggesting the high stability of the aqueous suspensions of NPs without using any additional stabilizers. The results of the physical–chemical studies of NPs demonstrated that the synthesis of CeO_2_ NPs via precipitation in the water–alcohol solutions enables the production of the NPs with controlled sizes and Ce^4+^/Ce^3+^ ratios.

### 3.2. Assessment of the Antiamyloidogenic Activity

**Figure 2:**
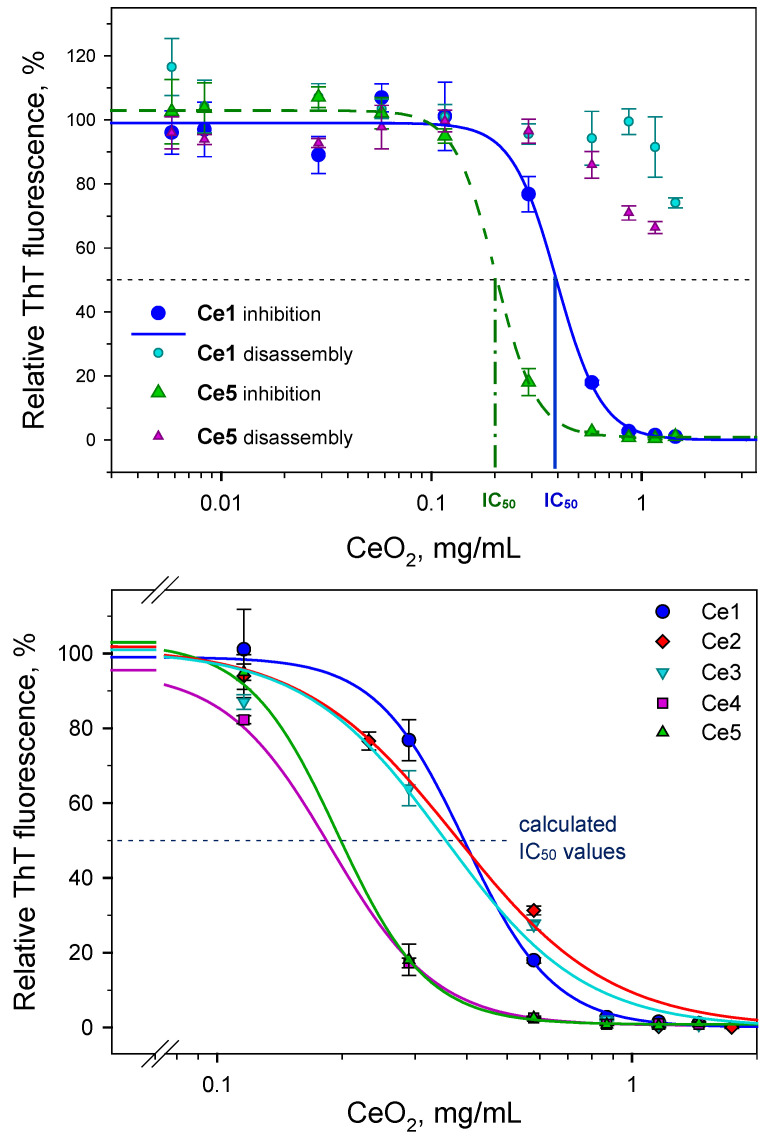
Concentration-dependent effects of CeO_2_ NPs on self-association of insulin monitored via ThT fluorescence assay. **Upper panel**—Ce1- and Ce5-NP-induced inhibition of insulin fibrillization and disassembly of preformed insulin fibrils. **Lower panel**—Detailed view on differences in the half-maximal inhibitory (IC_50_) values for all five studied CeO_2_ NPs. The antiamyloidogenic effect was quantified as a function of CeO_2_ NP concentrations ranging from 1:0.02 to 1:10 ratios (*w*/*w*) at a fixed 25 µM concentration of insulin/fibrils.

To perform a dose-dependent inhibition measurement, Ce1–Ce5 particles across a wide concentration range were added to 25 µM native insulin in NaCl solution and exposed to the conditions leading to the formation of amyloid structures as described in the Materials and Methods. The extent of insulin fibrillogenesis in the presence of CeO_2_ NPs was evaluated by fluorescence ThT assay based on increased fluorescence upon the binding of dyes to amyloid fibrils. The fluorescence intensity of control insulin samples (fibrils formed alone) was taken as 100% and the antiamyloid effect was quantified by normalizing a ThT fluorescence intensity of CeO_2_-NP-containing samples to the control. As documented in Figure 2, an increase in the CeO_2_ NPs concentration resulted in decreased fluorescence intensities, as presented for Ce1 (blue circles) and Ce5 (green triangles). The experimentally obtained data were further fitted using 4-parameter sigmoidal curves (Figure 2, upper panel). In general, a decrease in ThT fluorescence is interpreted as a decrease in the number of amyloid structures in the solution. Therefore, the data suggest that the antiamyloidogenic inhibition activity of the studied CeO_2_ particles is dose-dependent.

Moreover, sigmoidal decline curves enable quantification of the inhibiting activity by calculation of the half-maximal inhibiting IC_50_ values of CeO_2_ NPs. The calculated IC_50_ values for all 5 studied CeO_2_ NPs are listed in Table 1 and were found to be in the range of ~200 µg/mL to ~400 µg/mL. It appears that the calculated IC_50_ values exhibited a size-dependent tendency, i.e., the highest potential to inhibit insulin amyloid aggregation was observed for the smallest Ce5 particles, and on the contrary the weakest inhibition activity was shown for Ce1 particles. A size-dependent efficacy to alter amyloid aggregation was also observed for other types of nanoparticles, as documented for dextran-coated SPIONs (superparamagnetic iron oxide particles) [39]; plain-, positively- and negatively-coated SPIONs [40]; or modeled spherical Lennard–Jones particles [41]. L-glutathione-stabilized gold nanoparticles (AuNPs) differ in size and influence of Aβ fibrillization, whereby larger AuNPs accelerate fibrilization and small AuNPs significantly suppress the formation of fibrils, as observed by Gao et al. [42]. Similarly, Moore et al. emphasizes the important interplay between NP size and surface chemistry [43]. It is important to note that antiamyloid activity experiments have been performed in acidic conditions. Therefore, the zeta-potential of nanoparticles in pure NaCl solution as well as in the presence of native insulin, were measured (Table 1). At least two important conclusions can be made from the results. First, the NPs remained very stable, even at acidic pH, since zeta-potentials for CeO_2_ NPs in NaCl solution at pH 1.6, the range was from ~+33 up to ~+38 mV (Table 1). Second, a decrease in ζ-potential values in the presence of insulin indicates that the surface of CeO_2_ NPs is covered by insulin molecules. Thus, this may also affect the fibrillization of insulin. However, it should be noted that examined CeO_2_ NPs have rather low ability to disassemble pre-formed amyloid fibrils, as is shown in Figure 2 for two selected particles. The maximum disaggregation activity reached only 35–40% (Figure 2, upper panel).

**Figure 3:**
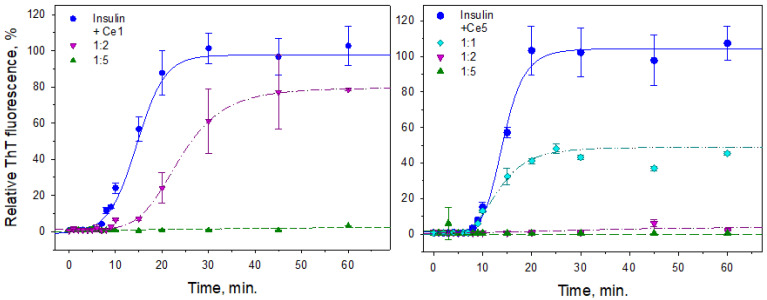
Time dependence of insulin fibrillization in the presence of CeO_2_ NPs evaluated by ThT assay. Ce1 (**left panel**) and Ce5 (**right panel**) particles were added to the freshly prepared insulin solution and samples were exposed to fibrillization conditions with and without CeO_2_ NP.

To obtain more detailed information about the process of inhibition, a kinetic study of insulin fibrillization alone and in the presence of all studied CeO_2_ NPs using the ThT assay was performed. In Figure 3, the time dependence of insulin fibrillization in the presence of the largest (Ce1, left panel) and smallest (Ce5, right panel) particles is shown. As expected, the fibrillization process exhibits a typical sigmoidal time dependency with four discrete phases: a first phase ~8–10 min long, a lag phase, and a ~20 min long elongation phase within fibrils, then finally the fibrillization culminated in a steady-state plateau phase when process reflected the presence of mature cross-β-rich amyloid fibrils. The presence of CeO_2_ NPs led to significantly altered kinetics and influenced the extent of insulin fibrillogenesis in a concentration-dependent manner. Figure 3 demonstrates the alterations in antiamyloid inhibition activity for two selected NPs, Ce1 and Ce5. At the protein-to-Ce1 nanoparticle ratio of 1:2 (mg/mg), a two-fold prolonged lag phase and moderate shallow *S*-curves were observed in comparison to the control insulin samples. The maximum ThT fluorescence intensity levels at the plateau phase reached ~80% of the control samples. Significant inhibition of the formation of amyloid structures in the presence of Ce1 was observed at the protein-to-nanoparticle ratio of 1:5, as documented by the pink symbols and pink curves (Figure 3, left panel). On the other hand, the smallest Ce5 particles characterized by lower IC_50_ values markedly affected the fibrillization, even at a 1:1 ratio (Figure 3, right panel, cyan symbols), when ThT fluorescence reached only 50% of control. Completely suppressed insulin fibrillization in the presence of Ce5 particles occurred at the protein-to-nanoparticle ratio of 1:2. The obtained results suggest that the antiamyloidogenic activity of CeO_2_ NPs arises from the inhibition of oligomerization and early stages of nucleation. A similar pattern of inhibition activity was also exhibited for CeO_2_ NPs prepared via precipitation from a reversal microemulsion [30]. The obtained results also suggested that the inhibition potential of newly prepared CeO_2_ NPs is most likely size-dependent. Smaller NPs more effectively bind to the ends of growing aggregates and more effectively inhibit fibrillization. However, the effects of the surface chemistry (represented by different Ce^4+^/Ce^3+^ ratios) cannot be excluded.

Recently, we demonstrated an inhibiting effect of cerium oxide nanoparticles synthesized by precipitation in a reversal microemulsion (CeO_2ME_ NPs) on insulin fibrillogenesis [35]. These NPs were about 4–6 nm in diameter [35] and characterized by the presence of ~28% of Ce^3+^ on the surface. Thus, CeO_2ME_ NPs combined the physical–chemical properties of Ce1 and Ce3 NPs. However, CeO_2ME_ NPs were 1.4- and 1.6-fold more effective in inhibiting insulin fibrillization, respectively, but less effective than smaller Ce4 and Ce5 NPs with higher percentages of Ce^3+^. The effects of cerium oxide particles on amyloid aggregation were also observed on ntrinsically disordered/natively unfolded proteins, including Aβ_1–42_ peptide, β_2_-microglobulin and α-synuclein [44,45,46]. However, information regarding the antiamyloidogenic effect of CeO_2_ NPs on globular protein amyloidogenesis is very limited [35,47]. Zand et al. demonstrated that the presence of ~30 nm CeO_2_ NPs led to a longer lag phase and a decreased α-synuclein fibrillization level [46]. The effects of the extension of the lag phase and suppression of the growth phase of lysozyme fibrillization in the presence of pure and surface-modified ~18 nm CeO_2_ NPs were also demonstrated by Samai et al. [47].

**Figure 4:**
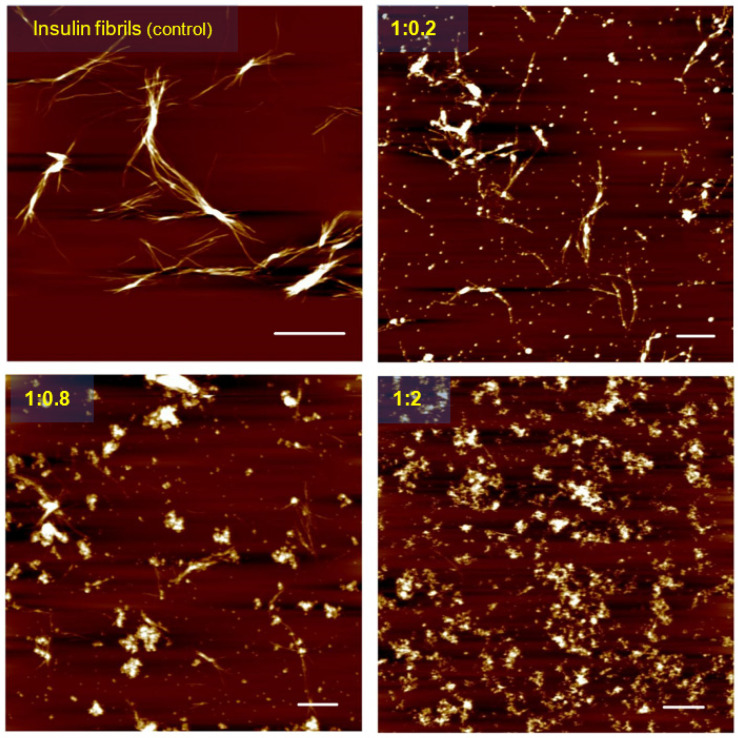
The AFM images of insulin amyloid fibrils formed alone (control) and in the presence of different concentrations of Ce2 (inhibition of amyloid aggregation). White scale bars represent 2 µm.

To verify the results obtained using ThT and ANS fluorescence assays, atomic force microscopy was applied to visualize the morphology of insulin amyloid structures formed in the presence and absence of different concentrations of CeO_2_ NPs. When incubated alone, insulin formed typical fibrillar structures. As documented in Figure 4, several micrometer-long and unbranched insulin fibrils showed a tendency to associate into larger complexes. The typical topological features of insulin amyloid fibrils were changed when fibrils were formed in the presence of nanoparticles. In Figure 4, representative scans of fibrils formed in the presence of Ce2 are presented. At low concentrations of Ce2 (protein-to-Ce2 ratio of 1:0.2, mg/mg), a lower amount of insulin fibrils can be observed. Further increases in Ce2 concentration led to noticeable changes in both the fibrils’ morphology and extent of fibrillization. Finally, fibrillization of insulin in the presence of Ce2 > 1:2 (mg/mg) was significantly inhibited, since the formation of mainly amorphous and sporadic amyloid-like aggregates was observed.

### 3.3. The Antioxidative Potential of CeO_2_ NPs

As a result of the low redox potential, the coexistence of the Ce^4+^/Ce^3+^ redox pair, and the presence of oxygen vacancies on the surface, cerium oxide nanoparticles exhibit enzyme-like (nanozymes) catalytic activities [48,49,50,51,52]. In this work, we evaluated the antioxidant activity of a set of Ce1–Ce5 particles by monitoring their SOD-like activities.

To assess the impacts of the size and redox state (oxidation state) on SOD’s mimetic activity, the set of prepared Ce1–Ce5 particles was tested using a commercial colorimetric SOD kit. The studied nanoparticles at a final concentration of 75 µg/mL were added to well plates with WST-1 reagent. The reaction was initiated by adding xanthine oxidase to generate superoxide radical anions. The nascent superoxide radicals reduced a water-soluble tetrazolium salt to formazan. The SOD catalyzed the dismutation of the superoxide anion into hydrogen peroxide and molecular oxygen, resulting in decreased WST-1 reduction as monitored by absorbance at 450 nm. From the calibration curve obtained using the SOD enzyme, the SOD-like activity of nanoparticles is expressed as units of SOD activity (Table 2).

Our results demonstrate well-defined valence- and size-dependent SOD-like activity. The highest SOD-like activity was observed for the smallest (~2.8 nm) nanoparticles with a large ratio of the surface area to volume, but more importantly with a high percentage of Ce^3+^ at the surface. The detailed mechanism of SOD’s mimetic activity of cerium oxide nanoparticles was proposed by Celardo et al. [49]. As described by Celardo, the stoichiometry of the reaction requires two superoxide anions reduced for each H_2_O_2_ molecule that is oxidized [49]. However, alternatively, the second molecule of H_2_O_2_ may oxidize the reduced Ce^3+^, as proposed previously [36,52,53], leading to the formation of Ce^4+^ and reduction of H_2_O_2_ to H_2_O. The last case represents a true catalase-like dismutation cycle [49]. If this mechanism is confirmed, CeO_2_ NPs may scavenge two abundant types of ROS as an endless redox machine. This redox machine can protect both organic molecules and living cells from the oxidative action of hydrogen peroxide, as was demonstrated for non-doped and terbium-doped CeF_3_ nanoparticles [50].

**Figure 5:**
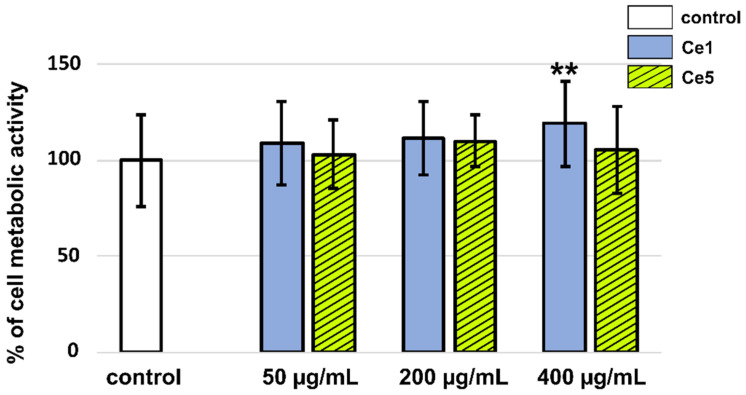
MTT assay of U87 MG cells in the absence (white columns) and presence of Ce1 (blue columns) or Ce5 (green hatched columns) nanoparticles at concentrations of 50, 200, and 400 µg/mL. The level of significance was evaluated with a one-way ANOVA test (** *p* < 0.01 in comparison to control).

CeO_2_ NPs possess enzyme-like properties that may affect the cell’s metabolic activity. Therefore, we evaluated the metabolic activity of cells in the presence of Ce1 and Ce5 using an MTT assay (Figure 5), which is a well-known indicator of cell viability, proliferation, and cytotoxicity. We did not detect significant changes in metabolic activity of U87 MG cells in the presence of CeO_2_ NPs, except a slight increase at the highest concentration (400 µg/mL) of Ce1 nanoparticles. The observed results were similar to those published previously [35].

**Figure 6:**
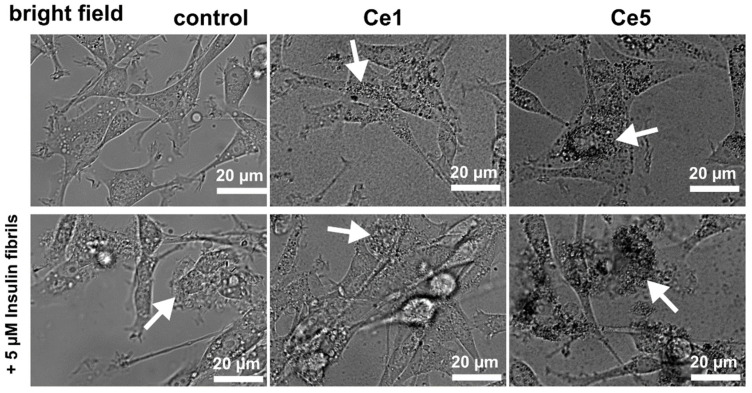
Representative bright-field images of U87 MG cells were subjected to 150 µg/mL of Ce1 or Ce5 nanoparticles and 5 µM insulin fibrils (second row) for 24 h. White arrows point to nanoparticle and fibril localization.

Protein misfolding and the subsequent aggregation are associated with many metabolic dysfunction diseases. Recently, the strong cytotoxicity of pathogenic protein aggregates, insulin included, was demonstrated in rat adrenal gland cells [54]. In the present work, the morphology of U87 MG cells in the presence and absence of insulin fibrils and CeO_2_ NPs was visualized using confocal microscopy. Bright-field images showed dark vesicles (~1.2 µm in diameter) localized in the cytosols of the studied cells (see white arrows in Figure 6). More contrasted spots of Ce5 NPs were found in the perinuclear areas. Regarding the size of CeO_2_ NPs (2.8–13.4 nm), the NPs localized in the vesicles are organized in clusters. These clusters were also observed in the extracellular space in the medium. The distribution of CeO_2_ NPs and vesicles in cells can be better recognized through inverted contrast (Figure S1).

The morphology of the cells with insulin fibrils was similar to the control cells. Insulin fibrils can be recognized in the images as deposits of predominately individual fibrils (3–6 µm) localized near the cell surface in the extracellular area. In contrast, the added CeO_2_ NPs were absorbed on the fibril surfaces, which led to the formation of larger bunches of fibrils (see white arrows denoted with “f” in Figure S1). The deposits of fibrils in the presence of Ce1 were 5–6 µm in diameter, while fibrils after absorption of Ce5 formed larger deposits of 8–15 µm in diameter. The fibrillar structures in the cell medium were stained with Hoechst and the differences in their size can be recognized in Figure S2. In addition, the vesicles with the CeO_2_ NPs in the cytosol and aggregates of CeO_2_ NPs in the extracellular area were observed as well.

We assume that nanoparticles, due to their aggregation, size, and vesicle localization, are preferentially transported into the cells by endocytosis. Lysosomes are the most important organelles in this type of transport. Vassie et al. reported in their study that the uptake of nanoceria into ovarian and colon cancer cells is energy-dependent and internalizes into the lysosomes [55]. In our experiments, CeO_2_ NPs were incubated for 24 h in cell culture media with serum proteins that could enable coating of the CeO_2_ NP surfaces, facilitate its uptake by U87 MG. In fact, Mazzolini et al. posed the hypothesis that serum-protein-enriched medium can enable the formation of protein coronas on nanoceria with proteins involved in endocytosis [56]. U87 MG cells express receptors that can control and mediate endocytosis [57].

**Figure 7:**
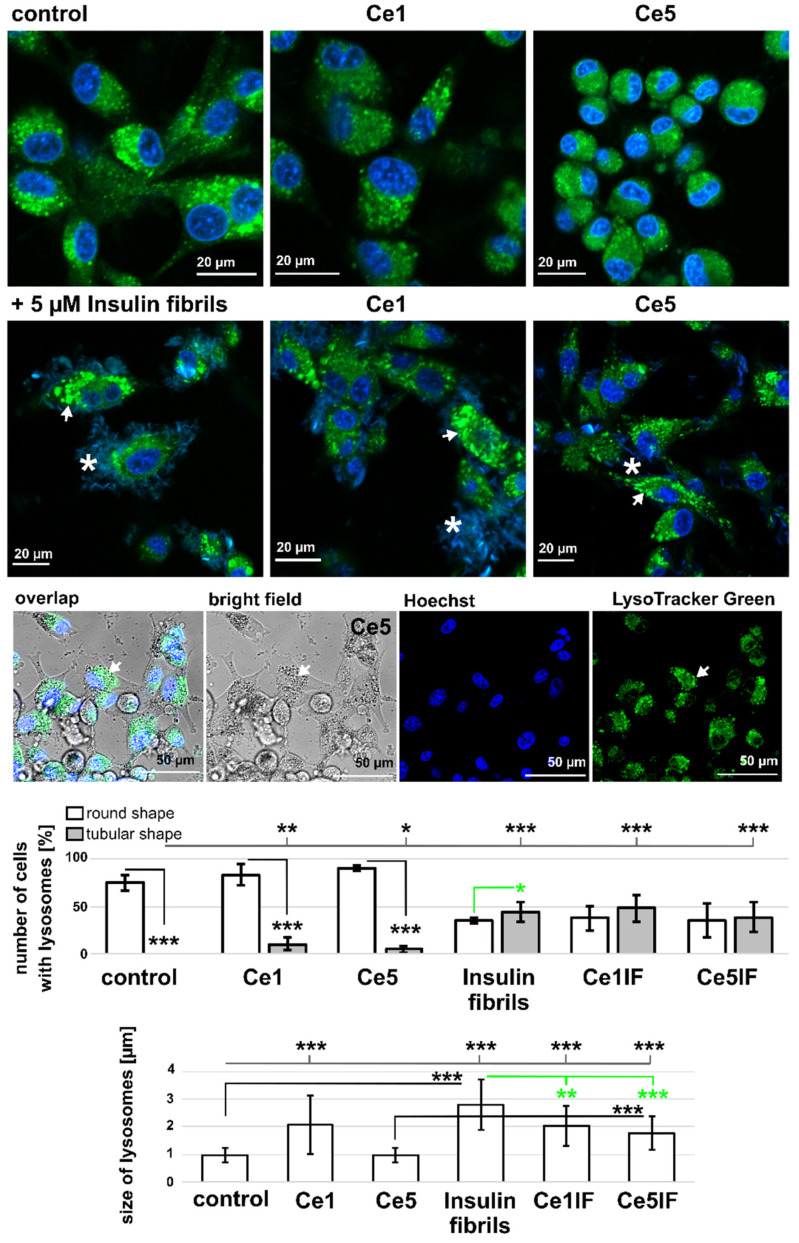
Representative fluorescent images of U87 MG cells labeled with LysoTracker Green (green, lysosomes) and Hoechst (blue, nuclei). The cells were subjected for 24 h to Ce1 and Ce5 nanoparticles at a concentration of 150 µg/mL and to 5 µM insulin fibrils (IF). Tubular-shaped lysosomes in the presence of insulin fibrils are denoted with white arrows (2nd row). Insulin fibrils adsorbed to the cell surface can be observed as blue fibers (white asterisks). A representative overlap image of a bright-field image with the fluorescence of Hoechst (blue)- and LysoTracker Green (green)-labeled cells in the presence of Ce5 NPs (3rd row). Co-localization of lysosomes and Ce5-NP-loaded vesicles is denoted with a white arrow. The numbers of cells with round (white columns) and tubular (grey columns) lysosomes were evaluated (400 cells for each studied case). The size of the lysosome was measured as the diameter of the lysosome. The level of significance was estimated with a one-way ANOVA test: * *p* < 0.05, ** *p* < 0.01, *** *p* < 0.001.

The distribution of lysosomes in U87 MG cells in the presence and absence of CeO_2_ NPs and insulin fibrils was detected with confocal fluorescence microscopy. Round-shaped lysosomes (green in Figure 7) were observed in control cells and cells subjected to Ce1 and Ce5 NPs. More lysosomes were found in cells treated with Ce1 than with Ce5 NPs. The size of lysosomes in the presence of Ce1 NPs was significantly larger (2.08 ± 1.06 µm in diameter) than in control and Ce5-NP-treated cells. Moreover, partial destabilization of lysosomes in the presence of Ce5 NPs caused diffused localization of Lysotracker Green fluorescence. Additionally, co-localization of the CeO_2_-NP-loaded vesicles and lysosomes can be seen in Figure 7 (3rd row). The application of insulin fibrils, which were applied together with nuclei labeled with Hoechst (blue in Figure 7), resulted in fusion and swelling of lysosomes. Significantly more cells with elongated lysosomes were detected in the presence of insulin fibrils. The number of lysosomes was less, but the organelles were significantly bigger (see Figure 7). In control cells, the size of the lysosomes was 0.97 ± 0.27 µm, while in the insulin-fiber-treated cells the size was 2.78 ± 0.9 µm in diameter. However, the application of CeO_2_ NPs rebutted this effect with the fission of lysosomes. The dynamics of lysosomal fission and fusion play important roles in endo- and exocytosis and in the detoxication of foreign particles in the cell. One of the proteins regulating lysosomal function and biogenesis is cathepsin B [58], the loss of which is attributed to lysosomal dysfunction [59]. Fluorescently immunolabelled U87 MG cells with cathepsin B (lysosomes), giantin (Golgi apparatus), and DAPI (nuclei) are presented in Figure S3. Giantin’s depicted Golgi apparatus assembly was composed of compact cisternae, which were surrounded by the number of round vesicles detected with cathepsin B. Elevated distribution of cathepsin B can be recognized in cells in the presence of CeO_2_ NPs and insulin fibrils. It should be noted that CeO_2_ NPs were also observed in the extracellular space (see an asterisk in Figure S3) after acetone fixation of cells. One should be aware of CeO_2_-NP-positive immunolabeling with cathepsin B and DAPI labeling of insulin fibrils. Bhoophathi et al. reported interplay between apoptosis and autophagy through the formation of autophagic vacuoles, induction of cathepsin B, and elevation of microtubule-associated protein light chain 3 (LC3) levels in secreted proteins, whereby the acidic and rich cysteine overexpressed primitive neuroectodermal tumor cells [60]. Song et al. in their work demonstrated that nanoceria coated with the organic surface can activate the lysosome autophagy system to detoxify human cervical cancer cells from nanoceria [61]. For this reason, we estimated the LC3B level in the U87 cells using the Western blot technique.

The presence of insulin fibrils in cell culture media increased LC3B levels in U87 MG cells (Figure S4). On the contrary, the application of CeO_2_ NPs decreased the LC3B level in cells. Two fractions, LC3B I and LC3B II, were recognized. Kabeya et al. reported that LC3B I is converted into LC3B II, which is integrated into the membranes during the formation of autophagic vesicles [62]. The ratio of LC3B II to LC3B I increases with autophagosomes formation. While Ce1 NPs decreased the LC3B II/LC3B I ratio in cells subjected to insulin fibrils, Ce5 NP application increased this ratio (Figure S4). The ratio was significantly higher in the absence than in the presence of insulin fibrils, but smaller than that of the natural polyphenol rottlerin (see Figure S5 in Supplementary Materials), a known autophagy inducer [63]. These results suggest that Ce5 NP application induces autophagy in glioma cells. Indeed, the fusion of lysosomes and cathepsin B observed in cells subjected to insulin fibrils may indicate autophagosome formation. However, the conversion of LC3B I into LC3B II was not observed (Figure S4). Moreover, a high level of cellular catalase was detected in these cells. It is well accepted that the cellular pool of ROS is constantly changing and balancing with the antioxidant system. The ROS balance is mainly governed by catalase, superoxide dismutase, and glutathione. The absence in the maturation of autophagic vesicles in cells subjected to insulin fibrils can be explained by the neutralization of ROS with catalase and catalase-like activity of CeO_2_ NPs. The application of CeO_2_ NPs further significantly decreased the catalase level. A clear difference between Ce1 and Ce5 NPs activities in cells was revealed. Yu et al. have shown that degrading the catalase can induce autophagy and cell death [64]. A significant reduction in catalase level and a high LC3B autophagic ratio was found in glioma cells in the presence of Ce5 NPs. SOD1 and thioredoxin levels in U87 MG cells were mainly affected by insulin fibrils (see Figure S6 in Supplementary Materials). Co-administration of CeO_2_ NPs reversed this effect.

**Figure 8:**
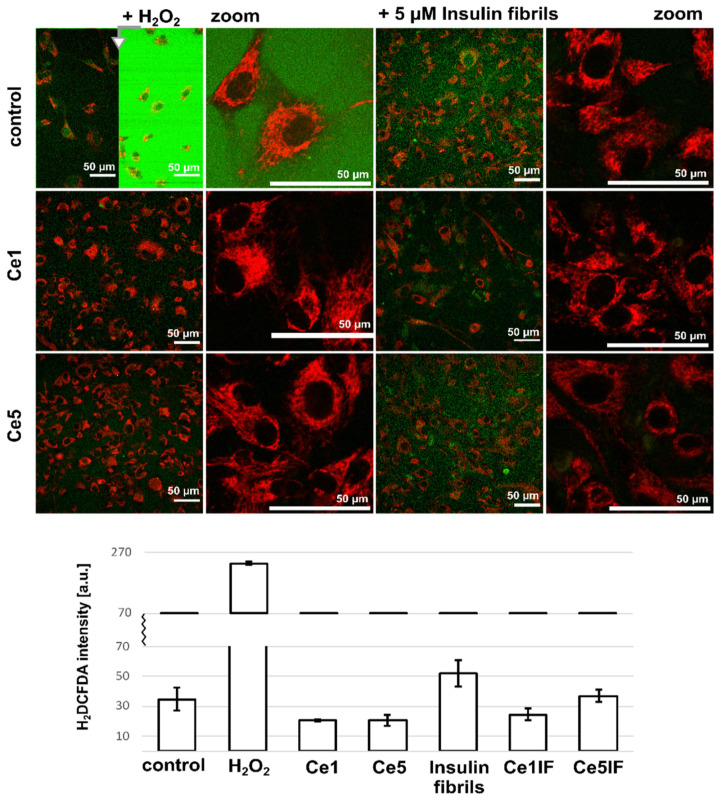
Representative fluorescence images of H_2_DCFDA (green) and TMRM (red, in mitochondria) in U87 MG cells. The cells were subjected for 24 h to Ce1 and Ce5 nanoparticles at 150 µg/mL and with 5 µM insulin fibrils. ROS production was induced with 100 µM H_2_O_2_. Fluorescence intensity levels of H_2_DCFDA were plotted into the histograms (under fluorescence images). Fluorescence images in the absence of TMRM are depicted in Figure S7.

**Figure 9:**
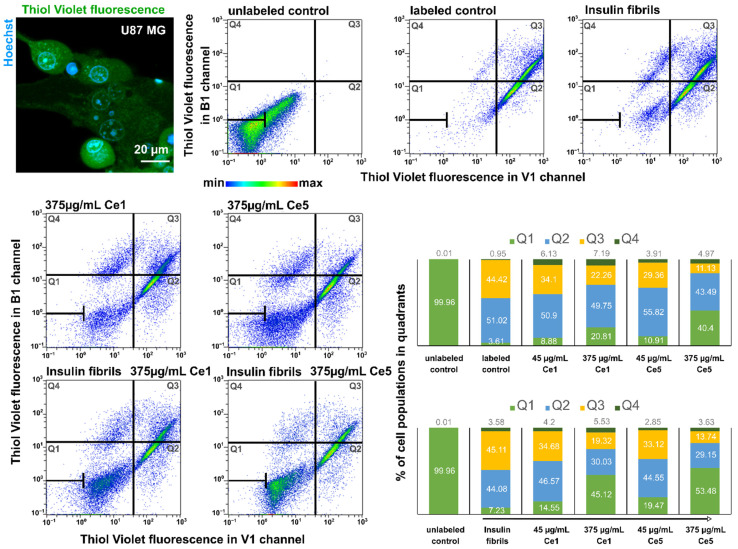
Reduced glutathione (GSH) levels in cells were monitored with ThiolTracker Violet fluorescence via flow cytometry. An illustrative fluorescence image of ThiolTracker Violet distribution in U87 MG cells is presented in the upper left panel. The cells were subjected to 5 µM insulin fibrils, and Ce1 and Ce5 nanoparticles at concentrations of 45 and 375 µg/mL. Cell populations in dot plots were divided into 4 quadrants (Q1–Q4). The numbers of cells and events are color-coded (blue—minima; red—maxima). Unlabeled cells were localized in Q1 (green histograms). Labeled untreated cell populations were identified in Q2 (blue histograms) and Q3 (yellow histograms). The quantification of cell populations is summarized in histograms (lower histograms—cells treated with insulin fibrils).

Cytotoxicity and oxidative stress induced by cerium oxide nanoparticles were observed in different types of cells and were shown to be shape-, size-, and concentration-dependent [65,66,67]. In the present work, oxidative stress in cells was monitored with H2DCFDA fluorescence, which increased after ROS production, e.g., after H_2_O_2_ administration (see Figure 8). Insulin fibrils present in the cell culture media increased the ROS production in cells, as well extracellularly (Figure 8 and Figure S7). The application of CeO_2_ NPs significantly decreased the oxidative stress in cells. Ce1′s antioxidant effect on cells with insulin fibrils was stronger than that of Ce5. Regarding the results obtained via Western blot analysis (Figures S4 and S6), the antioxidant effects of CeO_2_ NPs can be related to peroxide and superoxide production and the activity of catalase and superoxide dismutase. A negligible effect of CeO_2_ NPs and insulin fibrils was observed on thioredoxin levels detected in cells. A rich mitochondrial network was detected with TMRM in cells without decreased mitochondrial potential (Figure 9).

The reduction in ThiolTracker related to GSH can be explained similarly as for catalase as being due to the antioxidant-like activity of CeO_2_ NPs. One can hypothesize that Ce5 NPs can play the role of a Trojan horse that “convinces the cells” to take over oxidative stress control. However, instead of proliferation, cells will undergo autophagy.

## 4. Conclusions

The major goal of this work was to establish whether CeO_2_ NPs could be used in future experiments to prove or disprove the hypothesis that oxidative stress may play a role in the modulation of amyloidogenic protein self-assembly. Therefore, we systemically analyzed the bioactivity of synthesized CeO_2_ NPs. A set of CeO_2_ NPs with different sizes ranging from ~3 to 14 nm and various Ce^4+^/Ce^3+^ ratios at the surface was synthesized by precipitation in water–alcohol solutions with isopropanol as a component. The larger Ce1 and smaller Ce5 are characterized by having the lowest and highest percentages of Ce^3+^ ions at the surface, respectively.

Regardless of the size or Ce^4+^/Ce^3+^ ratio, all tested NPs strongly inhibited the formation of insulin amyloid aggregates in vitro. Among the NPs tested, the smaller Ce5 (d = 2.8 nm), which is also characterized by having the highest percentage of Ce^3+^ at the surface (47%), exhibited the strongest inhibition effect, as demonstrated by both ThT and ANS fluorescence assays. Only limited disaggregation of the studied NPs was observed. The synthesized CeO_2_ NPs exhibited well-defined, valence-dependent SOD-like properties.

CeO_2_ NPs were well tolerated by glioma cells. The NPs localized in acidic compartments of cells affected the biogenesis of lysosomes, which was enhanced by the introduction of insulin fibrils. This effect was attributed to autophagy and confirmed by Western blot analysis and immunostaining of autophagic protein LC3B. Such biological activity led to decreased GSH levels in cells, especially when NPs with a higher percentage of Ce^3+^ were administered. This indicates the ability of CeO_2_ NPs to modulate oxidative stress in a biological environment.

Overall, the results of the present study revealed the antiamyloid effect of cerium oxide nanoparticles synthesized by precipitation in water–alcohol solutions on insulin amyloid fibril formation. In addition, the NPs showed significant antioxidant activity by regulating the oxidant and antioxidant cell balance. Both the antiamyloid and antioxidant activity levels of NPs were size- or valence-dependent. Thus, CeO_2_ NPs show great potential for studying the interplay between oxidative stress and amyloid-related diseases and to prove or disprove the hypothesis that oxidative stress may play a role in the modulation of protein amyloid formation.

## Figures and Tables

**Table 1:** Experimentally determined IC_50_ values and surface properties of the studied Ce1–Ce5 NPs.

CeO_2_ NPs	Antiamyloid Inhibiting Activity IC_50_ in µg/mL		ζ-Potential [mV]		d_TEM_ [nm] ^(a)^ in H_2_O-IPA	*w* Ce^3+^ [%] ^(a)^ in H_2_O-IPA
	ThT	ANS	NaCl Solution	in the Presence of Insulin		
Ce1	400 ± 18	126 ± 4	33.4	24.9	13.4	28
Ce2	390 ± 15	140 ± 8	35.3	28.9	8.0	35
Ce3	355 ± 20	103 ± 4	37.8	25.3	5.6	40
Ce4	183 ± 9	70 ± 4	36.9	29.5	3.5	42
Ce5	198 ± 4	50 ± 1.5	35.7	28.1	2.8	47

Note: ^(a)^ described recently by Shlapa et al. [36]; d_TEM_ [nm] is the diameter of NPs determined by TEM; *w* Ce^3+^ [%] is the percentage of Ce^3+^ ions on the CeO_2_ surface.

**Table 2:** The SOD-like activity of CeO_2_ NPs monitored using an SOD kit.

CeO_2_ NPs	SOD-Like Activity of CeO_2_ NPs	
	Inhibition Rate %	U/mL
Ce1	12.5 ± 8.4	0.56 ± 0.047
Ce2	13.7 ± 8.1	0.61 ± 0.05
Ce3	13.9 ± 4.5	0.62 ± 0.028
Ce4	25 ± 3.6	1.18 ± 0.05
Ce5	33 ± 3.1	1.67 ± 0.052

## Data Availability

The datasets used or analyzed in this study are available from the corresponding author on reasonable request.

## Supplementary Materials

The following supporting information can be downloaded at: https://www.mdpi.com/article/10.3390/biomedicines10050942/s1, Material and Methods: Immuno-staining; Western blot; Figure S1: Representative bright-field images of U87 MG cells were subjected for 24 h to Ce1 and Ce5 nanoparticles at 150 µg/mL and with 5 µM insulin fibrils; Figure S2: Representative bright-field and fluorescence images of 5 µM insulin fibrils incubated for 24 h in cell culture medium with U87 MG cells and Ce1 and Ce5 nanoparticles at 150 µg/mL; Figure S3: Representative overlapped (in left) bright-field and fluorescence images of U87 MG cells immunolabeled with giantin (green) and DAPI (cyan), as well as fluorescence images (in right) of cathepsin B (cyan, Alexa Fluor 546), giantin (red, Alexa Fluor 488) and DAPI (yellow); Figure S4: Western blot analysis of catalase and LC3B levels in U87 MG cells; Figure S5: Western blot analysis of catalase and LC3B levels in U87 MG cells; S6: Western blot analysis of catalase (from Figure 5), SOD1, and thioredoxin levels in U87 MG cells; Figure S7: Representative fluorescence images of H_2_DCFDA (cyan) in U87 MG cells.

## References

[1] Chiti F., Dobson C.M. (2017). Protein Misfolding, Amyloid Formation, and Human Disease: A Summary of Progress over the Last Decade. Annu. Rev. Biochem..

[2] Iadanza M.G., Jackson M.P., Hewitt E.W., Ranson N.A., Radford S.E. (2018). A new era for understanding amyloid structures and disease. Nat. Rev. Mol. Cell Biol..

[3] Stefani M. (2004). Protein misfolding and aggregation: New examples in medicine and biology of the dark side of the protein world. Biochim. Biophys. Acta.

[4] Dobson C.M. (2017). The Amyloid Phenomenon and Its Links with Human Disease. Cold Spring Harb. Perspect. Biol..

[5] Lévy E., El Banna N., Baïlle D., Heneman-Masurel A., Truchet S., Rezaei H., Huang M.-E., Béringue V., Martin D., Vernis L. (2019). Causative Links between Protein Aggregation and Oxidative Stress: A Review. Int. J. Mol. Sci..

[6] Dahl J.-U., Gray M.J., Jakob U. (2015). Protein quality control under oxidative stress conditions. J. Mol. Biol..

[7] Tabner B.J., El-Agnaf O.M.A., German M.J., Fullwood N.J., Allsop D. (2005). Protein aggregation, metals and oxidative stress in neurodegenerative diseases. Biochem. Soc. Trans..

[8] Wang X., Wang W., Li L., Perry G., Lee H., Zhu X. (2014). Oxidative stress and mitochondrial dysfunction in Alzheimer’s disease. Biochim. Biophys. Acta.

[9] De Groot N.S., Burgas M.T. (2015). Is membrane homeostasis the missing link between inflammation and neurodegenerative diseases?. Cell. Mol. Life Sci..

[10] Reichmann D., Voth W., Jakob U. (2018). Maintaining a Healthy Proteome during Oxidative Stress. Mol. Cell.

[11] Samson A.L., Knaupp A.S., Kass I., Kleifeld O., Marijanovic E.M., Hughes V.A., Lupton C.J., Buckle A.M., Bottomley S.P., Medcalf R.L. (2014). Oxidation of an exposed methionine instigates the aggregation of glyceraldehyde-3-phosphate dehydrogenase. J. Biol. Chem..

[12] Carija A., Navarro S., de Groot N.S., Ventura S. (2017). Protein aggregation into insoluble deposits protects from oxidative stress. Redox Biol..

[13] Cheignon C., Jones M., Atrián-Blasco E., Kieffer E., Faller P., Collin F., Hureau C. (2017). Identification of key structural features of the elusive Cu–Aβ complex that generates ROS in Alzheimer’s disease. Chem. Sci..

[14] Cheignon C., Tomas M., Bonnefont-Rousselot D., Faller P., Hureau C., Collin F. (2018). Oxidative stress and the amyloid beta peptide in Alzheimer’s disease. Redox Biol..

[15] Ross C.A., Poirier M.A. (2005). Opinion: What is the role of protein aggregation in neurodegeneration?. Nat. Rev. Mol. Cell Biol..

[16] Cohen E., Bieschke J., Perciavalle R.M., Kelly J.W., Dillin A. (2006). Opposing activities protect against age-onset proteotoxicity. Science.

[17] Waugh D.F., Wilhelmson D.F., Commerford S.L., Sackler M.L. (1953). Studies of the Nucleation and Growth Reactions of Selected Types of Insulin Fibrils. J. Am. Chem. Soc..

[18] Brange J., Andersen L., Laursen E.D., Meyn G., Rasmussen E.J. (1997). Toward understanding insulin fibrillation. J. Pharm. Sci..

[19] Nielsen L., Frokjaer S., Carpenter J.F., Brange J. (2001). Studies of the structure of insulin fibrils by Fourier transform infrared (FTIR) spectroscopy and electron microscopy. J. Pharm. Sci..

[20] Dobson C.M. (2003). Protein folding and misfolding. Nature.

[21] Dische F.E., Wernstedt C., Westermark G.T., Westermark P., Pepys M.B., Rennie J.A., Gilbey S.G., Watkins P.J. (1988). Insulin as an amyloid-fibril protein at sites of repeated insulin injections in a diabetic patient. Diabetologia.

[22] Yumlu S., Barany R., Eriksson M., Röcken C. (2009). Localized insulin-derived amyloidosis in patients with diabetes mellitus: A case report. Hum. Pathol..

[23] Sie M.P.S., van der Wiel H.E., Smedts F.M.M., de Boer A.C. (2010). Human recombinant insulin and amyloidosis: An unexpected association. Neth. J. Med..

[24] Taguchi M., Takami S., Naka T., Adschiri T. (2009). Mechanism and Surface Chemical Characteristics of Dicarboxylic Acid-Modified CeO_2_ Nanocrystals Produced in Supercritical Water: Tailor-Made Water-Soluble CeO_2_ Nanocrystals. Cryst. Growth Des..

[25] Deshpande S., Patil S., Kuchibhatla S., Seal S. (2005). Size dependency variation in lattice parameter and valency states in nanocrystalline cerium oxide. Appl. Phys. Lett..

[26] Das S., Dowding J.M., Klump K.E., McGinnis J.F., Self W., Seal S. (2013). Cerium oxide nanoparticles: Applications and prospects in nanomedicine. Nanomedicine.

[27] Dowding J.M., Das S., Kumar A., Dosani T., McCormack R., Gupta A., Sayle T.X.T., Sayle D.C., von Kalm L., Seal S. (2013). Cellular interaction and toxicity depend on physicochemical properties and surface modification of redox-active nanomaterials. ACS Nano.

[28] Charbgoo F., Bin Ahmad M., Darroudi M. (2017). Cerium oxide nanoparticles: Green synthesis and biological applications. Int. J. Nanomed..

[29] Sun C., Li H., Chen L. (2012). Nanostructured ceria-based materials: Synthesis, properties, and applications. Energy Environ. Sci..

[30] Dowding J.M., Song W., Bossy K., Karakoti A., Kumar A., Kim A., Bossy B., Seal S., Ellisman M.H., Perkins G. (2014). Cerium oxide nanoparticles protect against A beta-induced mitochondrial fragmentation and neuronal cell death. Cell Death Differ..

[31] Nelson B.C., Johnson M.E., Walker M.L., Riley K.R., Sims C.M. (2016). Antioxidant cerium oxide nanoparticles in biology and medicine. Antioxidants.

[32] Banavar S., Deshpande A., Sur S., Andreescu S. (2021). Ceria nanoparticle theranostics: Harnessing antioxidant properties in biomedicine and beyond. J. Phys. Mater..

[33] Sharpe E., Frasco T., Andreescu D., Andreescu S. (2013). Portable ceria nanoparticle-based assay for rapid detection of food antioxidants (NanoCerac). Analyst.

[34] Ispas C., Njagi J., Cates M., Andreescu S. (2008). Electrochemical studies of ceria as electrode material for sensing and biosensing applications. J. Electrochem. Soc..

[35] Siposova K., Huntosova V., Shlapa Y., Lenkavska L., Macajova M., Belous A., Musatov A. (2019). Advances in the Study of Cerium Oxide Nanoparticles: New Insights into Antiamyloidogenic Activity. ACS Appl. Biol. Mater..

[36] Shlapa Y., Timashkov I., Veltruska K., Siposova K., Garcarova I., Musatov A., Solopan S., Kubovcikova M., Belous A. (2021). Structural and physical-chemical characterization of redox- active CeO_2_ nanoparticles synthesized by precipitation in water-alcohol solutions. Nanotechnology.

[37] Siposova K., Kozar T., Huntosova V., Tomkova S., Musatov A. (2019). Inhibition of amyloid fibril formation and disassembly of pre-formed fibrils by natural polyphenol rottlerin. Biochim. Biophys. Acta Proteins Proteom..

[38] Siposova K., Kozar T., Stupakova M., Musatov A. (2021). Complementary experimental and computational analysis of the effects of non-ionic detergents and phospholipids on insulin amyloid aggregation. Colloids Surf. B.

[39] Siposova K., Pospiskova K., Bednarikova Z., Safarik I., Safarikova M., Kubovcikova M., Kopcansky P., Gazova Z. (2017). The molecular mass of dextran used to modify magnetite nanoparticles affects insulin amyloid aggregation. J. Magn. Magn. Mater..

[40] Mahmoudi M., Quinlan-Pluck F., Monopoli M.P., Sheibani S., Vali H., Dawson K.A., Lynch I. (2013). Influence of the Physiochemical Properties of Superparamagnetic Iron Oxide Nanoparticles on Amyloid β Protein Fibrillation in Solution. ACS Chem. Neurosci..

[41] O’Brien E.P., Straub J.E., Brooks B.R., Thirumalai D. (2011). Influence of nanoparticle size and shape on oligomer formation of an amyloidogenic peptide. J. Phys. Chem. Lett..

[42] Gao G., Zhang M., Gong D., Chen R., Hua X., Sun T. (2017). The size-effect of gold nanoparticles and nanoclusters in the inhibition of amyloid-β fibrillation. Nanoscale.

[43] Moore K.A., Pate K.M., Soto-Ortega D.D., Lohse S., van der Munnik N., Lim M., Jackson K.S., Lyles V.D., Jones L., Glassgow N. (2017). Influence of gold nanoparticle surface chemistry and diameter upon Alzheimer’s disease amyloid-β protein aggregation. J. Biol. Eng..

[44] Wu W.H., Sun X., Yu Y.P., Hu J., Zhao L., Liu Q., Zhao Y.F., Li M. (2008). TiO_2_ Nanoparticles Promote Beta-Amyloid Fibrillation In Vitro. Biochem. Biophys. Res. Commun..

[45] Linse S., Cabaleiro-Lago C., Xue W.F., Lynch I., Lindman S., Thulin E., Radford S.E., Dawson K.A. (2007). Nucleation of Protein Fibrillation by Nanoparticles. Proc. Natl. Acad. Sci. USA.

[46] Zand Z., Khaki P.A., Salihi A., Sharifi M., Nanakali N.M.Q., Alasady A.A.B., Aziz M.F., Shahpasand K., Hasan A., Falahati M. (2019). Cerium oxide NPs mitigate the amyloid formation of α-synuclein and associated cytotoxicity. Int. J. Nanomed..

[47] Samai B., Basu A., Mati S.S., Bhattacharya S.C. (2019). Antiamyloid activity of functionalized cerium oxide nanoparticle on lysozyme fibrillation: Spectroscopic and microscopic investigation. Materialia.

[48] Celardo I., De Nicola M., Mandoli C., Pedersen J.Z., Traversa E., Ghibelli L. (2011). Ce^3+^ Ions Determine Redox-Dependent Anti-Apoptotic Effect of Cerium Oxide Nanoparticles. ACS Nano.

[49] Celardo I., Pedersen J.Z., Traversa E., Ghibelli L. (2011). Pharmacological potential of cerium oxide nanoparticles. Nanoscale.

[50] Shcherbakov A.B., Zholobak N.M., Baranchikov A.E., Ryabova A.V., Ivanov V.K. (2015). Cerium fluoride nanoparticles protect cells against oxidative stress. Mater. Sci. Eng. C.

[51] Baldim V., Bedioui F., Mignet N., Margaillc I., Berret J.F. (2018). The enyzme-like catalytic activity of cerium oxide nanoparticles and its dependency on Ce^3+^ surface area concentration. Nanoscale.

[52] Heckert E.G., Karakoti A.S., Seal S., Self W.T. (2008). The role of cerium redox state in the SOD mimetic activity of nanoceria. Biomaterials.

[53] Das M., Patil S., Bhargava N., Kang J.F., Riedel L.M., Seal S., Hickman J.J. (2007). Auto-catalytic Ceria Nanoparticles Offer Neuroprotection to Adult Rat Spinal Cord Neurons. Biomaterials.

[54] Lee S., Choi M.C., Al Adem K., Lukman S., Lee T.Y.K. (2020). Aggregation and Cellular Toxicity of Pathogenic or Non-pathogenic Proteins. Sci. Rep..

[55] Vassie J.A., Whitelock J.M., Lord M.S. (2017). Endocytosis of cerium oxide nanoparticles and modulation of reactive oxygen species in human ovarian and colon cancer cells. Acta Biomater..

[56] Mazzolini J., Weber R.J.M., Chen H.S., Khan A., Guggenheim E., Shaw R.K., Chipman J.K., Viant M.R., Rappoport J.Z. (2016). Protein Corona Modulates Uptake and Toxicity of Nanoceria via Clathrin-Mediated Endocytosis. Biol. Bull..

[57] Huntosova V., Buzova D., Petrovajova D., Kasak P., Nadova Z., Jancura D., Sureau F., Miskovsky P. (2012). Development of a new LDL-based transport system for hydrophobic/amphiphilic drug delivery to cancer cells. Int. J. Pharm..

[58] Qi X., Man S.M., Malireddi R.K.S., Karki R., Lupfer C., Gurung P., Neale G., Guy C.S., Lamkanfi M., Kanneganti T.D. (2016). Cathepsin B modulates lysosomal biogenesis and host defense against *Francisella novicida* infection. J. Exp. Med..

[59] Cermak S., Kosicek M., Mladenovic-Djordjevic A., Smiljanic K., Kanazir S., Hecimovic S. (2016). Loss of Cathepsin B and L Leads to Lysosomal Dysfunction, NPC-Like Cholesterol Sequestration and Accumulation of the Key Alzheimer’s Proteins. PLoS ONE.

[60] Bhoopathi P., Chetty C., Gujrati M., Dinh D.H., Rao J.S., Lakka S. (2010). Cathepsin B facilitates autophagy-mediated apoptosis in SPARC overexpressed primitive neuroectodermal tumor cells. Cell Death Differ..

[61] Song S., Lee S.S., Savini M., Popp L., Colvin V.L., Segatori L. (2014). Ceria Nanoparticles Stabilized by Organic Surface Coatings Activate the Lysosome-Autophagy System and Enhance Autophagic Clearance. ACS Nano.

[62] Kabeya Y., Mizushima N., Ueno T., Yamamoto A., Kirisako T., Noda T., Kominami E., Ohsumi Y., Yoshimori T. (2000). LC3, a mammalian homologue of yeast Apg8p, is localized in autophagosome membranes after processing. EMBO J..

[63] Song K.S., Kim J.S., Yun E.J., Kim Y.R., Seo K.S., Park J.H., Jung Y.J., Park J.I., Kweon G.R., Yoon W.H. (2008). Rottlerin induces autophagy and apoptotic cell death through a PKC-delta-independent pathway in HT1080 human fibrosarcoma cells: The protective role of autophagy in apoptosis. Autophagy.

[64] Yu L., Wan F., Dutta S., Welsh S., Liu Z.H., Freundt E., Baehrecke E.H., Lenardo M. (2006). Autophagic programmed cell death by selective catalase degradation. Proc. Natl. Acad. Sci. USA.

[65] Kumari M., Singh S.P., Chinde S., Rahman M.F., Mahboob M., Groover P. (2014). Toxicity Study of Cerium Oxide Nanoparticles in Human Neuroblastoma Cells. Int. J. Toxicol..

[66] Forest V., Leclerc L., Hochepied J.F., Trouvé A., Sarry G., Pourchez J. (2017). Impact of cerium oxide nanoparticles shape on their in vitro cellular toxicity. Toxicol. In Vitro.

[67] Gallucci N., Vitiello G., Di Girolamo R., Imbimbo P., Monti D.M., Tarallo O., Vergara A., Krauss I.R., Paduano L. (2021). Towards the Development of Antioxidant Cerium Oxide Nanoparticles for Biomedical Applications: Controlling the Properties by Tuning Synthesis Conditions. Nanomaterials.

