# Supplementary material for: Dual-Functional Antioxidant and Antiamyloid Cerium Oxide Nanoparticles Fabricated by Controlled Synthesis in Water-Alcohol Solutions
Source: Biomedicines. 2022 Apr 19;10(5):942. doi: 10.3390/biomedicines10050942 (PMC9138294; doi:10.3390/biomedicines10050942)
Supplement: Supplementary file 1 [file biomedicines-10-00942-s001.zip › biomedicines-1662374-supplementary.pdf]

biomedicines

MDPI

Article

Dual-Functional Antioxidant and Antiamyloid Cerium Oxide Nanoparticles Fabricated by Controlled Synthesis in Water–Alcohol Solutions

Katarina Siposova [1,*,#], Veronika Huntosova [2,*,#], Ivana Garcarova [1], Yuliia Shlapa [3], Illia Timashkov [3], Anatolii Belous [3] and Andrey Musatov [1]

[1] Department of Biophysics, Institute of Experimental Physics, Slovak Academy of Sciences, Watsonova 47, 040 01 Kosice, Slovakia; garcarova@saske.sk (I.G.); musatov@saske.sk (A.M)
[2] Center for Interdisciplinary Biosciences, Technology and innovation park, P.J. Safarik University in Kosice, Jesenna 5, 041 54 Kosice, Slovakia;
[3] Vernadskii Institute of General and Inorganic Chemistry of NAS of Ukraine; Palladina ave., 32/3403142, Kyiv, Ukraine; yuliashlapa@ukr.net (Y.S.); timashkov@ukr.net (I.T.); agrbilous@ukr.net (A.B.)
* Correspondence: siposova@saske.sk (K.S.); veronika.huntosova@upjs.sk (V.H.)
[#] Both authors (K.S. and V.H.) contributed equally to this work.

Copyright: © 2022 by the authors. Licensee MDPI, Basel, Switzerland. This article is an open access article distributed under the terms and conditions of the Creative Commons Attribution (CC BY) license (https://creativecommons.org/licenses/by/4.0/).

Material and Methods

Immuno-staining

Cells were fixed after the treatments with ice-cold acetone (Centralchem, Bratislava, Slovakia) for 5 min at –20 °C. Cells were washed with ice-cold phosphate-buffered saline (PBS, Sigma-Aldrich, Darmstadt, Germany) and blocked in 5% bovine serum albumin (BSA, Sigma-Aldrich, Darmstadt, Germany) in PBS at 25 °C for 1 hour. Subsequently, cells were incubated with primary antibodies diluted in 5% BSA with anti-giantin (ab80864, Abcam, Cambridge, UK) and anti-cathepsin B (H-5) antibodies (sc-365558, Santa Cruz Biotechnology Santa Cruz, CA, USA) for 1 hour at 25 °C. After incubation, the cells were washed with ice-cold PBS. Further, the secondary antibodies conjugated to Alexa Fluor 488 (ab150077, Abcam, Cambridge, UK) or Alexa Fluor 546 (a11030, ThermoFisher Scientific, USA) and diluted in 1% BSA were applied to the cells for 1 hour at 25 °C. A mounting medium with 4′,6-diamidino-2-phenylindole (DAPI, ab104139, Abcam, Cambridge, UK) was utilized to stabilize the fluorescence signal and counterstain the nuclei. Fluorescence of secondary antibodies detection parameters: Alexa Fluor 488, excitation at 488 nm and emission of 500–540 nm; Alexa Fluor 546, excitation at 555 nm and emission > 560 nm; DAPI, excitation at 405 nm and emissions of 450 ± 40 nm.

Western blot

Western blot analysis of oxidative stress defense (catalase, smooth muscle actin, SOD1, TRX) and LC3B levels in U87 MG cells was performed with anti-LC3B ab221794 (1:3000) antibodies (Abcam, UK), while oxidative stress defense was analyzed with ab179843 Western blot cocktail (1:1000). Cell lysates were collected in RIPA buffer (150 mM NaCl, 1% Triton X-100, 0.5% sodium deoxycholate, 0.1% sodium dodecyl sulfate, 20 mM Tris buffer saline at pH 8.0) with inhibitor cocktail (2x 1:100, HaltTM Protease and Phosphatase Inhibitor Cocktail, ThermoFisher Scientific, USA) from cells incubated for 24 h with insulin fibrils, Ce1, and Ce5. Proteins were separated via electrophoresis, transferred onto a nitrocellulose membrane, and immunodetected with the Western Breeze Chromogenic Kit (ThermoFisher Scientific, USA) according to the supplier protocol. The optical densities of visualized bands were calculated using ImageJ software

(National Institutes of Health, USA). The level of significance was evaluated with a one-way ANOVA test (*$p<0.05$, **$p<0.01$, ***$p<0.001$).

Supplementary Figures and Captions

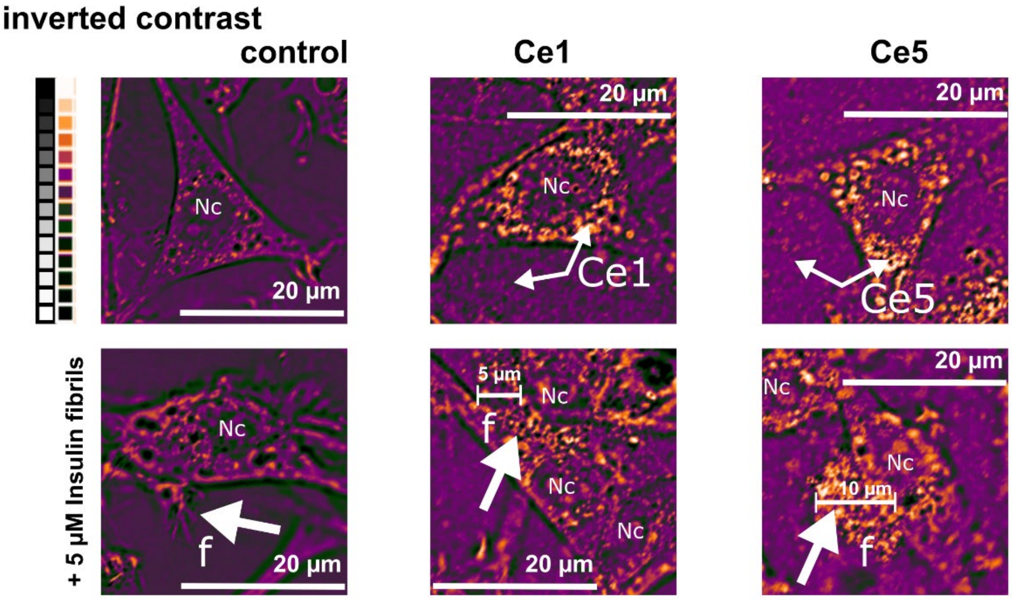

Figure S1. Representative bright-field images of U87 MG cells were subjected for 24 h to Ce1 and Ce5 nanoparticles at 150 μg/mL and with 5 μM insulin fibrils. White arrows point to nanoparticle and fibril localization. Images were converted via inverted contrast with the corresponding color code to recognize the most scattered particles inside cells. CeO_2 NPs and insulin fibrils can be identified in orange. CeO_2 NPs formed grainy surfaces in the extracellular space. Nc—nucleus; f—fibrils.

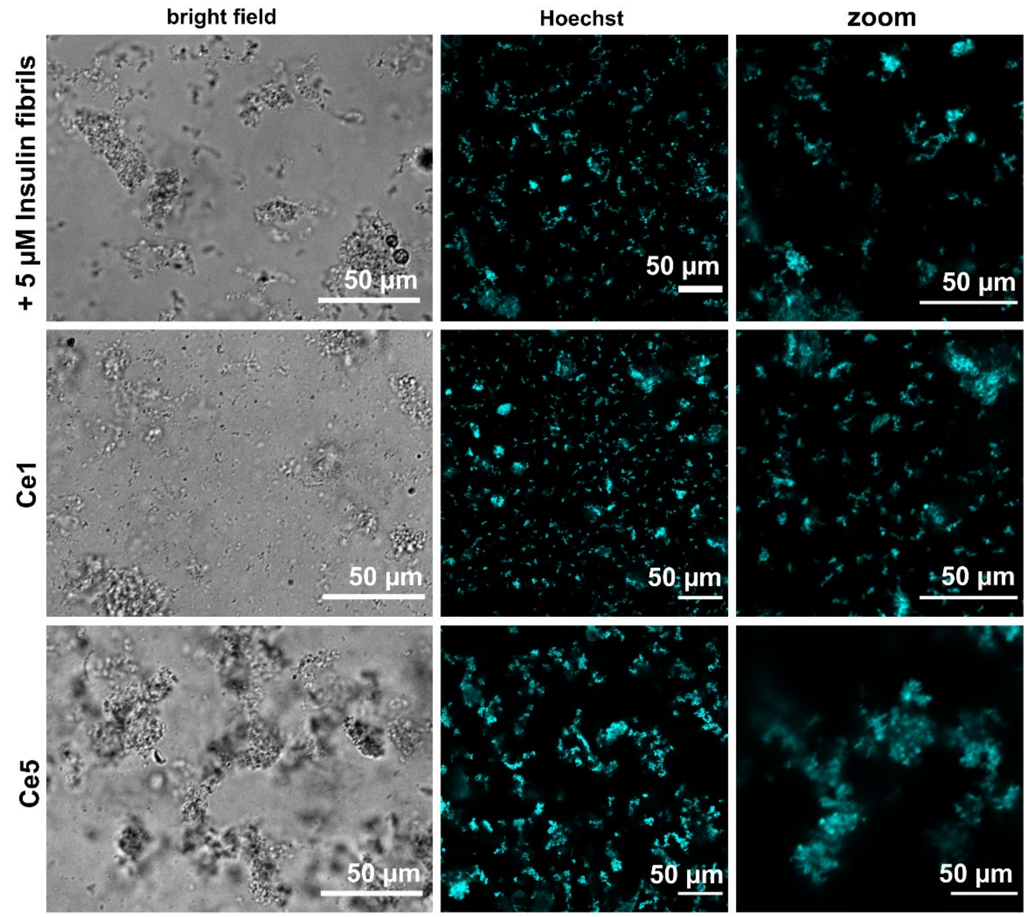

Figure S2. Representative bright-field and fluorescence images of 5 µM insulin fibrils incubated for 24 h in cell culture medium with U87 MG cells and Ce1 and Ce5 nanoparticles at 150 µg/mL. Cell cultivation medium was aspired and labeled with Hoechst.

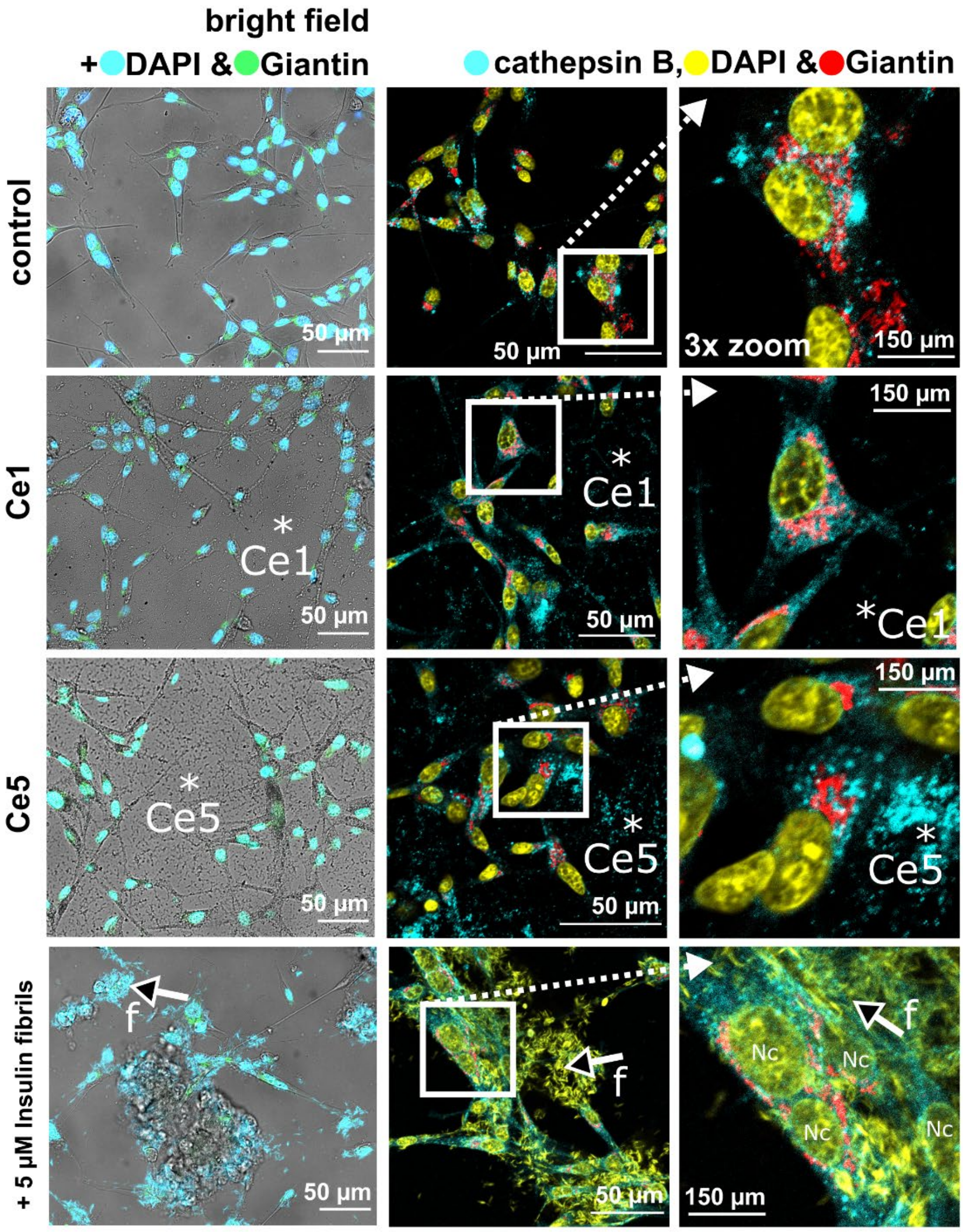

Figure S3. Representative overlapped (in left) bright-field and fluorescence images of U87 MG cells immunolabeled with giantin (green) and DAPI (cyan), as well as fluorescence images (in right) of cathepsin B (cyan, Alexa Fluor 546), giantin (red, Alexa Fluor 488) and DAPI (yellow). The cells were subjected for 24 h to Ce1 and Ce5 nanoparticles at 150 µg/mL with 5 µM insulin fibrils. Insulin fibrils can be recognized as cyan (right) and yellow (left) fibers and are denoted with "f" and arrows. The nanoparticles can be partially recognized in cyan and are denoted with an asterisk. Scale bar represents 50 µm. Nc—nucleus; f—fibrils. Selected areas were magnified to better recognize the specific localization of immunolabeling.

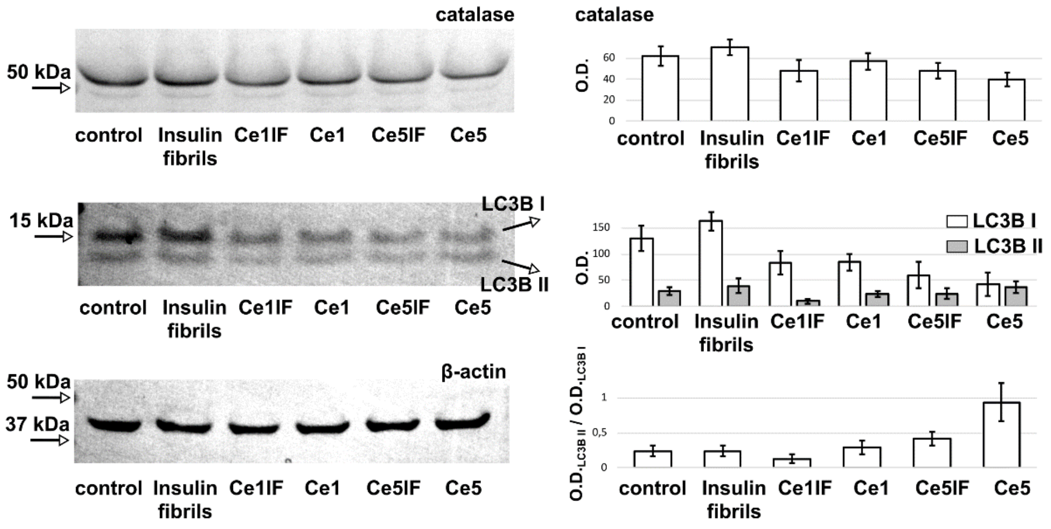

Figure S4. Western blot analysis of catalase and LC3B levels in U87 MG cells. β-Actin was detected as the housing protein. Optical densities (O.D.) are plotted as histograms. The LC3B II/LC3B I ratio is related to autophagy. The cells were subjected for 24 h to Ce1 and Ce5 nanoparticles at 150 μg/mL with 5 μM insulin fibrils.

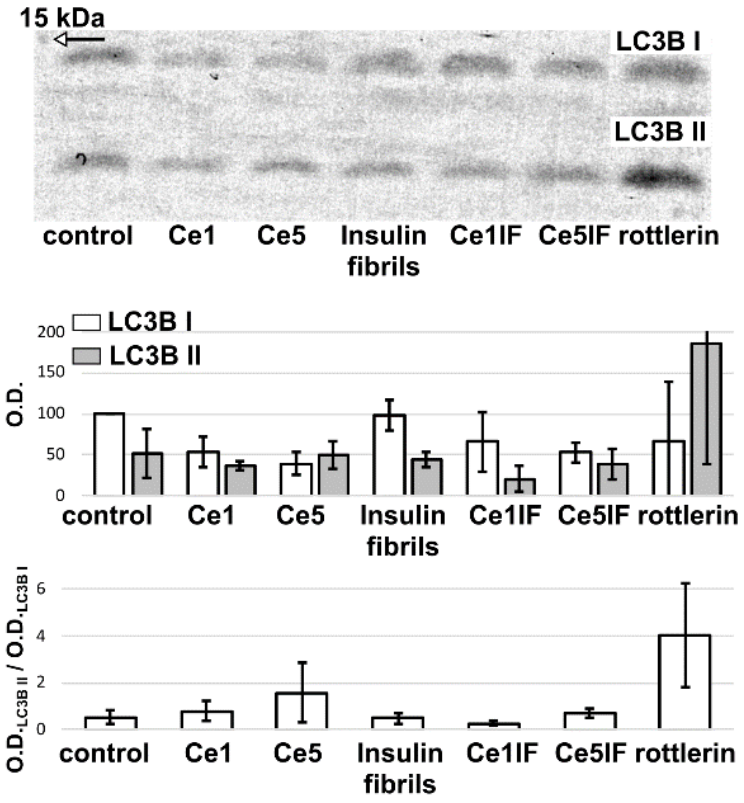

Figure S5. Western blot analysis of catalase and LC3B levels in U87 MG cells. β-Actin was detected as the housing protein (Figure 5). Optical densities (O.D.) are plotted as histograms. The LC3B II/LC3B I ratio is related to autophagy. The cells were subjected for 24 h to Ce1 and Ce5 nanoparticles at 150 μg/mL with 5 μM insulin fibrils (IF) and 10 μM rottlerin for 1 h as a positive control to autophagy.

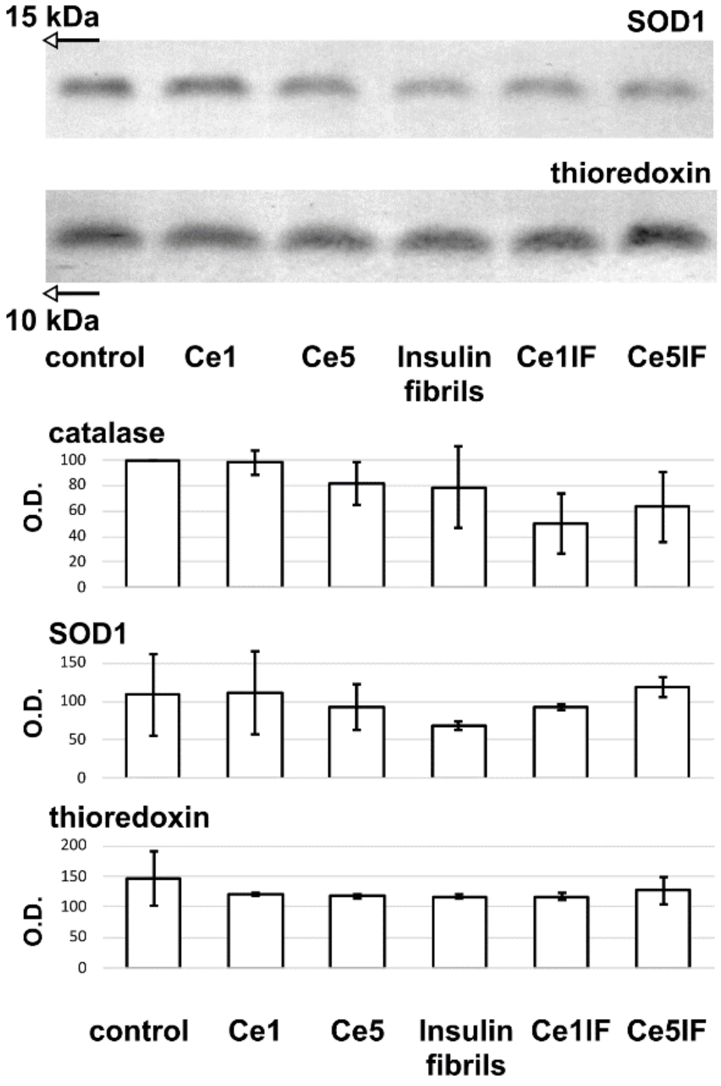

Figure S6. Western blot analysis of catalase (from Figure 5), SOD1, and thioredoxin levels in U87 MG cells. β-Actin was detected as the housing protein, as is presented in Figure 5. Optical densities (O.D.) are plotted in histograms. The cells were subjected for 24 h to Ce1 and Ce5 nanoparticles at 15 μg/mL with 5 μM insulin fibrils (IF).

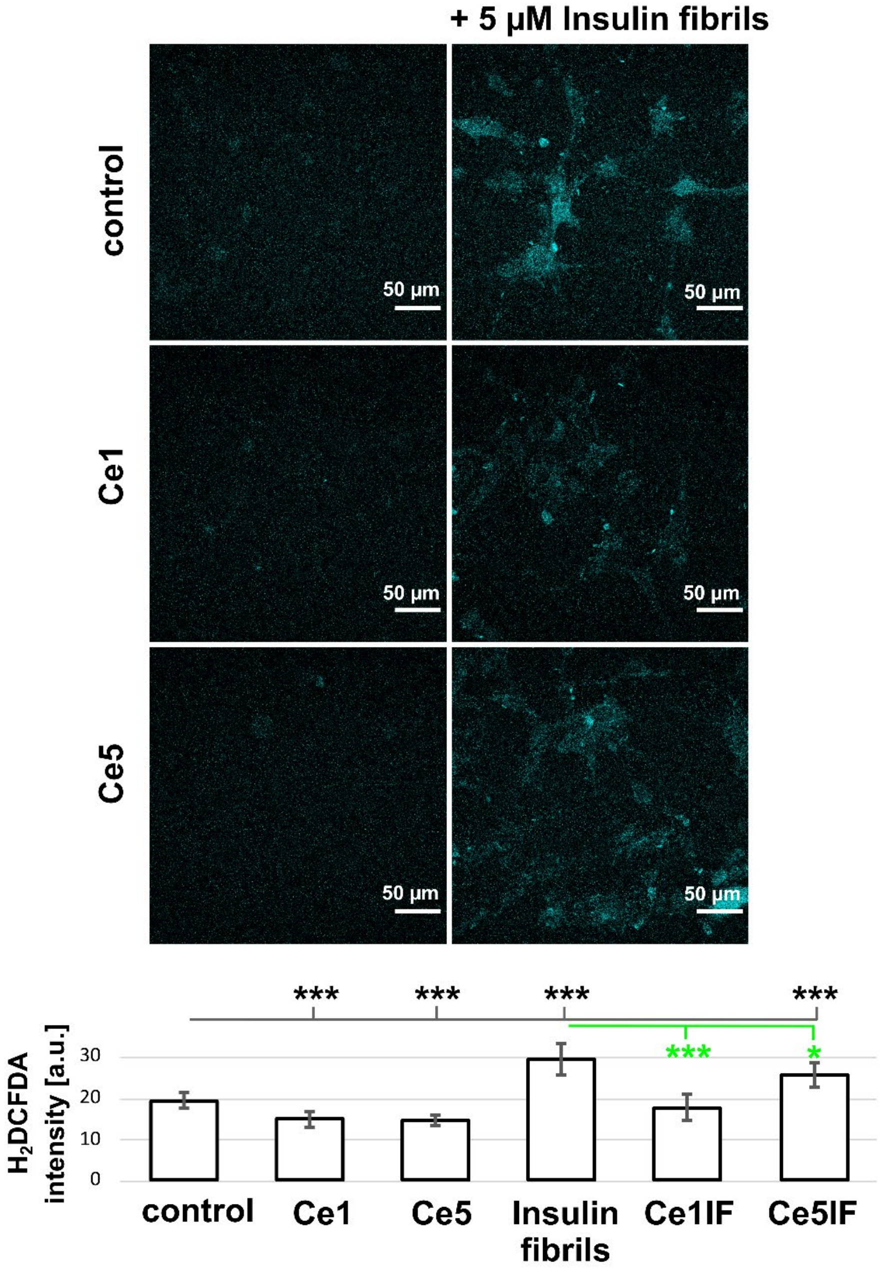

Figure S7. Representative fluorescence images of H_2DCFDA (cyan) in U87 MG cells. The cells were subjected for 24 h to Ce1 and Ce5 nanoparticles at 150 µg/mL with 5 µM insulin fibrils (IF). Fluorescence intensity levels of H_2DCFDA are plotted as histograms (under the images). Significant differences were calculated with ANOVA as *$p<0.05$, **$p<0.01$, ***$p<0.001$.
